# Use of a Dynamic Genetic Testing Approach for Childhood-Onset Epilepsy

**DOI:** 10.1001/jamanetworkopen.2019.2129

**Published:** 2019-04-12

**Authors:** Jorune Balciuniene, Elizabeth T. DeChene, Gozde Akgumus, Edward J. Romasko, Kajia Cao, Holly A. Dubbs, Surabhi Mulchandani, Nancy B. Spinner, Laura K. Conlin, Eric D. Marsh, Ethan Goldberg, Ingo Helbig, Mahdi Sarmady, Ahmad Abou Tayoun

**Affiliations:** 1 Division of Genomic Diagnostics, Department of Pathology and Laboratory Medicine, The Children's Hospital of Philadelphia, Philadelphia, Pennsylvania; 2 Division of Neurology, The Children's Hospital of Philadelphia, Philadelphia, Pennsylvania; 3 Department of Pathology and Laboratory Medicine, Perelman School of Medicine, University of Pennsylvania, Philadelphia; 4 Department of Neurology, Perelman School of Medicine, University of Pennsylvania, Philadelphia; 5 now with Department of Genomics, Al Jalila Children’s Specialty Hospital, Dubai, United Arab Emirates

## Abstract

**Importance:**

Although genetic testing is important for bringing precision medicine to children with epilepsy, it is unclear what genetic testing strategy is best in maximizing diagnostic yield.

**Objectives:**

To evaluate the diagnostic yield of an exome-based gene panel for childhood epilepsy and discuss the value of follow-up testing.

**Design, Setting, and Participants:**

A case series study was conducted on data from clinical genetic testing at Children’s Hospital of Philadelphia was conducted from September 26, 2016, to January 8, 2018. Initial testing targeted 100 curated epilepsy genes for sequence and copy number analysis in 151 children with idiopathic epilepsy referred consecutively by neurologists. Additional genetic testing options were offered afterward.

**Exposures:**

Clinical genetic testing.

**Main Outcomes and Measures:**

Molecular diagnostic findings.

**Results:**

Of 151 patients (84 boys [55.6%]; median age, 4.2 years [interquartile range, 1.4-8.7 years]), 16 children (10.6%; 95% CI, 6%-16%) received a diagnosis after initial panel analysis. Parental testing for 15 probands with inconclusive results revealed de novo variants in 7 individuals (46.7%), resulting in an overall diagnostic yield of 15.3% (23 of 151; 95% CI, 9%-21%). Twelve probands with nondiagnostic panel findings were reflexed to exome sequencing, and 4 were diagnostic (33.3%; 95% CI, 6%-61%), raising the overall diagnostic yield to 17.9% (27 of 151; 95% CI, 12%-24%). The yield was highest (17 of 44 [38.6%; 95% CI, 24%-53%]) among probands with epilepsy onset in infancy (age, 1-12 months). Panel diagnostic findings involved 16 genes: *SCN1A* (n = 4), *PRRT2* (n = 3), *STXBP1* (n = 2), *IQSEC2* (n = 2), *ATP1A2*, *ATP1A3*, *CACNA1A*, *GABRA1*, *KCNQ2*, *KCNT1*, *SCN2A*, *SCN8A*, *DEPDC5*, *TPP1*, *PCDH19*, and *UBE3A* (all n = 1). Exome sequencing analysis identified 4 genes: *SMC1A*, *SETBP1*, *NR2F1*, and *TRIT1*. For the remaining 124 patients, analysis of 13 additional genes implicated in epilepsy since the panel was launched in 2016 revealed promising findings in 6 patients.

**Conclusions and Relevance:**

Exome-based targeted panels appear to enable rapid analysis of a preselected set of genes while retaining flexibility in gene content. Successive genetic workup should include parental testing of select probands with inconclusive results and reflex to whole-exome trio analysis for the remaining nondiagnostic cases. Periodic reanalysis is needed to capture information in newly identified disease genes.

## Key Points

**Question:**

What genetic testing approach is most useful in maximizing diagnostic yield for children with idiopathic epilepsy?

**Findings:**

In this case series study of 151 patients referred for genetic epilepsy testing from a single academic tertiary hospital, the overall diagnostic yield was 17.9%. An initial exome-based 100-gene panel contributed 10.6%, while parental testing and reflex to exome analysis added 4.7% and 2.7%, respectively, and analysis expansion to 13 recently reported genes uncovered promising findings in 6 patients.

**Meaning:**

Exome-based panels may be a useful genetic testing option for children with idiopathic epilepsy, with parental testing being informative in establishing a definitive diagnosis.

## Teaser

This case series evaluates an exome-based gene panel approach to maximize diagnostic yield for children with idiopathic epilepsy.

## Introduction

Epilepsies are among the most common neurologic disorders, with an estimated prevalence of 6 in 1000 people worldwide.^1^ Epilepsies encompass a heterogeneous group of clinical entities with diverse causes and outcomes. Although up to a third of epilepsy cases are due to acquired insults, such as birth trauma, brain injury, or a tumor, the remainder are attributable to genetic factors and include epilepsies with monogenic and complex inheritance.^2,3^

Monogenic forms of seizure disorders tend to manifest earlier in life. Their clinical spectrum is broad, ranging from benign, self-limited epilepsies; epilepsies due to inborn errors of metabolism; epilepsies with other neurodevelopmental issues, such as autism or intellectual disability; and early-onset, severe epileptic encephalopathies.^4,5,6^ Early establishment of a specific diagnosis is necessary for providing an accurate prognosis and recurrence risk as well as optimizing management and treatment options. However, high genetic heterogeneity and phenotypic pleiotropy make it challenging to clinically differentiate between genetically distinct epilepsies. Pathogenic variants in numerous genes may lead to indistinguishable epilepsies, and variation in the same gene can result in epilepsies with differing presentation, both in severity and comorbidity manifestation.^2,4,6,7,8^ Therefore, genetic testing often serves as a useful tool in finding a definitive cause and concluding the diagnostic process.

Pathogenic variants in the epilepsies can be diverse, ranging from large copy number variants (CNVs) to simple DNA sequence variants. Over the past decade, the number of genes implicated in epilepsy and the repertoire of genetic tests offered have grown significantly.^9,10^ In addition to traditional cytogenetic and microarray-based tests, comprehensive next-generation sequencing (NGS) panels, exome, and genome NGS tests are available for genetic epilepsy testing.^6,11^ Each of those tests comes with its own advantages and limitations in terms of detectable genetic variant type, diagnostic yield, costs, and turnaround time. Although clinicians are challenged with navigating complex clinical algorithms for choosing the best genetic workup for a specific patient and with test result interpretation,^6,10^ clinical laboratories are urged to devise comprehensive testing options, keep testing costs low and turnaround times short, and find solutions for maximizing diagnostic yield while staying abreast of the pace of gene discovery.

To address these diverse clinical and economic demands, we have developed an exome sequencing (ES)-based test for the pediatric epilepsy diagnosis focusing on an initial analysis of 100 epilepsy genes. The ES-based strategy enables easy reflex to analysis of the full exome for cases with a negative panel outcome and offers flexibility in modifying the panel’s gene content in response to new gene discoveries or clinical demands. Herein, we report on evaluation of the clinical test usefulness for a cohort of 151 consecutive patients with childhood-onset epilepsy and discuss strategies for improving diagnostic yield.

## Methods

### Patients

This study cohort included 151 consecutive, unrelated patients referred by neurologists from a single academic tertiary medical center for genetic testing of childhood epilepsy between September 26, 2016, and January 8, 2018. All but 1 patient were tested using a comprehensive, exome-based epilepsy panel. The main indication for testing was epilepsy of unknown source in patients in whom epilepsy was the primary disease phenotype. The clinical patient data presented in this study are based on medical records available at the time of the test order. All patient samples were deidentified for this report. The study was reviewed by the institutional review board of Children’s Hospital of Philadelphia and was determined to meet exemption criteria with waiver of informed consent. This study followed the reporting guideline for case series.

### Gene Curation

The panel included 100 genes selected for targeted testing based on literature reports implicating variants in these genes with nonsyndromic epilepsy or apparently nonsyndromic, when epilepsy was the first presenting or most prominent feature. The strength of evidence for each gene’s association with disease was at least moderate based on an internal review using the Clinical Genome Consortium gene validity criteria.^12,13^ Recently discovered genes with limited evidence were also included in anticipation of additional evidence supporting their potential association with epilepsy.

### Comprehensive Exome-Based Sequencing Panel

Genomic DNA was obtained from peripheral blood or other patient tissues following standard DNA extraction protocols and was prepared for ES using previously described procedures.^14^ An in-house bioinformatics pipeline^15^ was used for NGS data analysis, including variant filtration and annotation. Only variants in the epilepsy panel regions of interest were retained (eMethods 1 in the Supplement). The filtration process captured known pathogenic variants, as well as rare variants with a potential association with protein function or RNA splicing. Rare variants included (1) variants with minor allele frequency less than 1% if previously reported in the Human Gene Mutation Database, (2) variants with minor allele frequency less than 0.1% in genes associated with a dominant forms of epilepsy, and (3) variants with minor allele frequency less than 0.57% in genes associated with recessive forms of epilepsy. The minor allele frequency cutoffs were calculated as previously described.^15^ Variants were then filtered based on their frequencies in the Exome Aggregation Consortium database.

Exon-level CNVs were called from ES coverage data by an in-house pipeline, using ExomeDepth.^16^ Coverage assessment and criteria for ad hoc Sanger sequencing fill-in have been described previously.^15^ More information about the ES-based and rapid STAT Epilepsy Panel can be found in eMethods 1 and eMethods 2 in the Supplement.

Genes included in the analysis of the ES epilepsy panel were *ALDH7A1*, *ALG13*, *ARHGEF9*, *ARX*, *ASAH1*, *ATP1A2*, *ATP1A3*, *CACNA1A*, *CACNA1D*, *CASK*, *CDKL5*, *CERS1*, *CHD2*, *CHRNA2*, *CHRNA4*, *CHRNB2*, *CLN3*, *CLN5*, *CLN6*, *CLN8*, *CNKSR2*, *CSTB*, *CTSD*, *DEPDC5*, *DNM1*, *DYNC1H1*, *EEF1A2*, *EFHC1*, *EPM2A*, *FOLR1*, *FOXG1*, *GABRA1*, *GABRB3*, *GABRG2*, *GNAO1*, *GOSR2*, *GRIN1*, *GRIN2A*, *GRIN2B*, *HCN1*, *HDAC4*, *HNRNPU*, *IQSEC2*, *KCNA1*, *KCNA2*, *KCNB1*, *KCNC1*, *KCNJ10*, *KCNQ2*, *KCNQ3*, *KCNT1*, *KCTD7*, *LGI1*, *MECP2*, *MEF2C*, *MFSD8*, *NHLRC1*, *PCDH19*, *PIGA*, *PIGO*, *PIGT*, *PLCB1*, *PNKP*, *PNPO*, *POLG*, *PPT1*, *PRICKLE1*, *PRRT2*, *PURA*, *QARS*, *RELN*, *RYR3*, *SCARB2*, *SCN1A*, *SCN1B*, *SCN2A*, *SCN8A*, *SIK1*, *SLC12A5*, *SLC13A5*, *SLC25A22*, *SLC2A1*, *SLC35A2*, *SLC6A1*, *SLC6A8*, *SPTAN1*, *ST3GAL3*, *STX1B*, *STXBP1*, *SYN1*, *SYNGAP1*, *SZT2*, *TBC1D24*, *TPP1*, *TSC1*, *TSC2*, *WDR45*, *WWOX*, *UBE3A*, and *ZEB2*. Gene identification numbers are available in the eTable in the Supplement.

### Variant Classification and Determination of Diagnostic Outcome

Rare sequence variants or CNVs were classified in accordance with the framework of the standards and guidelines published by the American College of Medical Genetics and Genomics.^17^ A result was considered diagnostic if a clinically significant variant (pathogenic or likely pathogenic) was identified in an autosomal-dominant gene or if there were 2 clinically significant variants identified in *trans* for autosomal-recessive genes. A negative result was concluded if no reportable variants were identified. All other results were considered inconclusive. Parental testing was offered free-of-charge for panel-inconclusive cases that had reportable variants in autosomal-dominant genes or 2 variants in autosomal-recessive genes. A subset of inconclusive reports was considered likely diagnostic and included cases that had 1 or more heterozygous variant in autosomal-dominant gene or 2 heterozygous variants in recessive genes that were rare or absent from large population studies, such as gnomAD,^18^ and that affected critical protein regions, such as ion channel domains, or those that had de novo variants in genes with limited clinical validity.

### Statistical Analysis

Categorical variables were summarized using proportions with 95% CIs provided where applicable. Median and interquartile range (IQR) were provided for continuous variables. A χ^2^ test of independence was performed using online tools^19^ to evaluate the significance of diagnostic yield across different patient groupings. For age at seizure onset group comparison, the early childhood and older groups were combined into 1 group; for seizure type group comparison, patients with febrile and unknown type of seizures were excluded; for brain magnetic resonance imaging findings and family history group comparisons, patients with undetermined magnetic resonance imaging findings or unknown family history were excluded. Williams correction for small sample size was applied where applicable. *P* < .01 was considered statistically significant. A tool available online was used for data analysis.^19^

## Results

### Cohort Description

**Figure 1.**
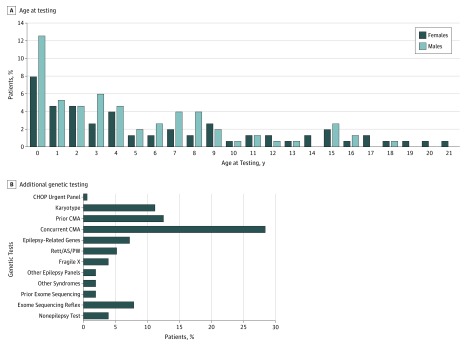
Cohort Demographics and Genetic Testing Data A, Age at testing and sex distribution. B, Additional genetic testing received by the patients. CHOP Urgent includes Rapid Epilepsy Sequence and Deletion/Duplication (STAT) Panel. Epilepsy-related genes include *SCN1A*, *SCN1B*, *GABRG2*, *ARX*, *CNTNAP2*, *TSC1/2*, *CHRNA4*, *CHRNB2*, and mitochondrial DNA. Other syndromes include Coffin-Lowry, X-linked mental retardation, and microcephaly. Rett/AS/PW indicates testing for Rett, Angelman, and Prader-Willi syndromes, and includes *MECP2* sequencing and deletion/duplication testing, CDKL5 sequencing, and atypical Rett syndrome testing. Prior exome sequencing arrays were performed at other diagnostic laboratories. Non-epilepsy testing includes genetic tests for thrombosis, ketotic hypoglycemia, ichthyosis, connexin 26, and tumor. CMA indicates chromosomal microarray analysis.

The patient cohort included 151 children and young adults with epilepsy with relatively even sex distribution (67 [44.4%] female and 84 [55.6%] male). Age at testing ranged between 0 and 22 years, with median age at testing of 4.2 years (IQR, 1.4-8.7 years) (Figure 1A). At the time of requisition, 69 patients (45.7%) were noted to manifest additional neurodevelopmental features, including developmental delay, learning disability, speech delay, and/or behavioral issues (referred to as seizure plus) (Table 1). Median age at seizure onset was 1.83 years (IQR, 0.5-3.75 years). Focal seizures were reported in 61 children (40.4%), followed by 49 children (32.5%) with generalized seizures and 26 children (17.2%) with mixed-type seizures. Magnetic resonance imaging findings were noted in 58 patients (38.4%); however, the findings were considered noncontributory by the patients’ neurologists and included reports such as atrophy, nonspecific white matter changes, or decreased myelination.

**Table 1.** Testing Outcome Stratified by Cohort Clinical Characteristics

Cohort	Patients, No. (% Category)			Category Total (% All Cohort)	*P* Value
	With Diagnostic Findings (n = 27)	With Likely Diagnostic and Inconclusive Findings	No Reportable Findings		
Age at seizure onset					
Neonatal (0-1 mo)	1 (12.5)	6 (75.0)	1 (12.5)	8 (5.3)	<.001^a^
Infancy (1 mo-1 y)	17 (38.6)	16 (36.4)	11 (25.0)	44 (29.1)	
Toddler (1-3 y)	8 (16.0)	28 (56.0)	14 (28.0)	50 (33.1)	
Early childhood (3-5 y)	0	12 (50.0)	12 (50.0)	24 (15.9)	
Middle childhood (6-11 y)	1 (5.3)	11 (57.9)	7 (36.8)	19 (12.6)	
Adolescent (12-18 y)	0	4 (66.7)	2 (33.3)	6 (4.0)	
Phenotype					
Seizure only	12 (14.6)	43 (52.4)	27 (32.9)	82 (54.3)	.52
Seizure plus^b^	15 (21.7)	34 (49.3)	20 (29.0)	69 (45.7)	
Seizure type					
Focal	12 (19.7)	30 (49.2)	19 (31.1)	61 (40.4)	.25
Generalized	6 (12.2)	27 (55.1)	16 (32.7)	49 (32.5)	
Mixed	9 (35)	10 (38)	7 (27)	26 (17.2)	
Febrile	0	1 (100)	0	1 (0.06)	
Unknown	0	9 (64.3)	5 (35.7)	14 (9.3)	
Brain MRI					
With findings^c^	15 (25.9)	24 (41.4)	19 (32.8)	58 (38.4)	.19
No findings	12 (15.4)	43 (55.1)	23 (29.5)	78 (51.7)	
Not determined	0	10 (66.7)	5 (33.3)	15 (9.9)	
Family history					
Positive	7 (14.0)	30 (60.0)	13 (26.0)	50 (33.1)	.19
Negative	20 (22.7)	39 (44.3)	29 (33.0)	88 (58.3)	
Unknown	0	8 (61.5)	5 (38.5)	13 (8.6)	

Abbreviation: MRI, magnetic resonance imaging.

^a^ Williams correction applied.

^b^ Includes patients presenting with developmental and behavioral phenotypes, such as developmental delay, developmental regression, speech or language delay, attention-deficit/hyperactivity disorder or autism.

^c^ Includes any abnormality identified by MRI.

All cases but 1 were tested by the exome-based epilepsy panel. One patient, originally referred to the exome panel, was instead expedited, per clinician’s request, for sequencing using a smaller, medically actionable rapid epilepsy panel (eMethods 2 in the Supplement).

### Additional Genetic Testing

Seventy-eight of the 151 patients (51.7%) had at least 1 additional genetic test done besides the epilepsy panel, for a total of 132 additional tests. Sixty-two patients (41.1%) had chromosomal microarray analysis (CMA), which was ordered at the clinician’s discretion mainly for patients with the seizure plus phenotype (Table 1). Forty-three patients (28.5%) had the CMA test performed concurrently with the panel and 19 patients (12.6%) had it performed before the epilepsy panel test.

Prior karyotype testing was performed on 17 patients (11.3%). Fifteen patients (9.9%) underwent ES: 3 in another laboratory before referral and 12 who were reflexed to ES after a nondiagnostic panel result. Additional genetic testing included analysis for single epilepsy genes (11 [7.3%]); other epilepsy panels (3 [2.0%]); fragile X (6 [4.0%]); Rett, Angelman, or Prader-Willi syndromes (8 [5.3%]); other neurologic syndromes (3 [2.0%]); or non–epilepsy-related conditions (6 [4.0%]) (Figure 1B).

Genetic testing done before or concurrently with the panel yielded significant findings in 5 patients. Three patients had trisomies identified by karyotype (1 had Klinefelter syndrome [XXY] and 2 had trisomy 21) that were not considered explanatory for their seizures and, therefore, were referred for epilepsy panel testing. The panel testing revealed a heterozygous *PCDH19* pathogenic variant in the patient with Klinefelter syndrome (patient 5) (Table 2), explaining his epilepsy.^20^ The other 2 patients (patients 11 and 15) had recurrent microdeletions (16p11.2 and 22q11.22q11.23, respectively) identified by concurrent CMA. The 16p11.2 deletion, which includes the *PRRT2* gene, was also detected using ES coverage data from this patient.

**Table 2.** Molecular Findings

Patient No.	Genomic Coordinates	Gene	Transcript	cDNA	Protein Effect	Zygosity	Classification	Inheritance	Parental Origin	Diagnosis by
1	Chr1:160106162	*ATP1A2*	NM_000702.3	c.2563 + 2T>C		Het	Likely pathogenic	AD	Maternal	Panel
2	ChrX:53280248	*IQSEC2*	NM_001111125.2	c.1510C>T	p.Gln504*	Hem	Likely pathogenic	XLD	Unknown	Panel
3	ChrX:53279758-53279776	*IQSEC2*	NM_001111125.2	c.1983_1999del	p.Leu662fs	Het	Likely pathogenic	XLD	Unknown	Panel
4	Chr20:62038729	*KCNQ2*	NM_172107.2	c.1888-1G>A		Het	Likely pathogenic	AD	Unknown	Panel
5	ChrX:99662890	*PCDH19*	NM_001184880.1	c.706C>T	p.Pro236Ser	Het	Likely pathogenic	XL	Unknown	Panel
6	Chr22:32098112-32270694	*PRR14L, DEPDC5*		22q12.2 - q12.3 deletion		Het	Likely pathogenic	AD	Unknown	Panel
7	Chr16:29824948-29824950	*PRRT2*	NM_001256442.1	c.577delG	p.Glu193Lysfs*36	Het	Pathogenic	AD	Unknown	Panel
8	Chr2:166850723-166850726	*SCN1A*	NM_001165963.1	c.4783_4784delCT	p.Leu1595fs	Het	Pathogenic	AD	Unknown	Panel
9	Chr2:166905395	*SCN1A*	NM_001202435.1	c.1028 + 1G>T		Het	Pathogenic	AD	Unknown	Panel
10	Chr2:166848438	*SCN1A*	NM_001165963.1	c.5347G>A	p.Ala1783Thr	Het	Pathogenic	AD	Unknown	Panel
11	Chr16:29595483-30198151	*PPRT2*^a^		16p11.2 deletion		Het	Pathogenic	AD	Unknown	Panel
12	Chr16:29652999-30194001	*PPRT2*^b^		16p11.2 deletion		Het	Pathogenic	AD	Unknown	Panel
13	Chr9:130430431dup	*STXBP1*	NM_003165.3	c.867dup	p.Ala290Serfs*24	Het	Pathogenic	AD	Unknown	Panel
14	Chr9:130430439	*STXBP1*	NM_003165.3	c.875G>A	p.Arg292His	Het	Pathogenic	AD	Unknown	Panel
15^c^	Chr11:6638271; Chr11:6638385	*TPP1*	NM_000391.3	c.622C>T; c.509-1G>C	p.Arg208*; p.?	Compound het	Pathogenic; pathogenic	AR	Both	Panel
16^c^	Chr9:138651532	*KCNT1*	NM_020822.2	c.862G>A	p.Gly288Ser	Het	Pathogenic	AD	De novo, recurrent	Panel
17	Chr19:42482319	*ATP1A3*	NM_001256214.1	c.1829G>A	p.Arg610His	Het	Likely pathogenic	AD	Likely de novo	Parental testing
18	Chr19:13372340	*CACNA1A*	NM_023035.2	c.4186G>A	p.Val1396Met	Het	Likely pathogenic	AD	Likely de novo	Parental testing
19	Chr5:161309591	*GABRA1*	NM_000806.5	c.587A>G	p.Tyr196Cys	Het	Likely pathogenic	AD	Likely de novo	Parental testing
20	Chr2:166848464	*SCN1A*	NM_001165963.1	c.5321T>C	p.Phe1774Ser	Het, mosaic	Likely pathogenic	AD	De novo	Parental testing
21	Chr2:166231468	*SCN2A*	NM_001040142.1	c.4246C>T	p.Leu1416Phe	Het	Likely pathogenic	AD	likely de novo	Parental testing
22	Chr12:52200543	*SCN8A*	NM_014191.3	c.5273T>C	p.Val1758Ala	Het	Likely pathogenic	AD	De novo	Parental testing
23	Chr15:25584341	*UBE3A*	NM_000462.3	c.2550_2570dup	p.Glu851_Lys857dup	Het	Likely pathogenic	Imprinted	Likely de novo, unknown	Parental testing
24	Chr5:92921095	*NR2F1*	M_005654.5	c.366C>G	p.Cys122Trp	Het	Likely pathogenic	AD	De novo	ES trio
25	Chr18:42531889	*SETBP1*	NM_015559.2	c.2584G>A	p.Glu862Lys	Het	Pathogenic	AD	De novo	ES trio
26	ChrX:53440181	*SMC1A*	NM_006306.3	c.615 + 1G>C		Het, mosaic	Pathogenic	XLD	De novo	ES trio
27^d^	Chr1:40319721-40319723; 40319730	*TRIT1*	NM_017646.5	c.334delC; c.326T>C;	p.Arg112fs; p.Ile109Thr	Compound het	VUS; VUS	AR	Both	ES trio
**Reanalysis Findings**										
28	Chr22:40036960	*CACNA1I*	NM_021096.3	c.829A>G	p.Ile277Val	Het	VUS	AD	Unknown	NA
29	Chr2:149267668	*MBD5*	NM_018328.4	c.4377T>A	p.His1459Gln	Het	VUS	AD	Unknown	NA
30	Chr2:166020387	*SCN3A*	NM_001081677.1	c.619G>C	p.Val207Leu	Het	VUS	AD	Unknown	NA
31	Chr2:166025265	*SCN3A*	NM_006922.3	c.454G>A	p.Asp152Asn	Het	VUS	AD	Unknown	NA
32	Chr6:118015256	*NUS1*	NM_138459.4	c.604A>G	p.Arg202Gly	Het	VUS	AD	Unknown	NA
33	Chr3:132394747	*UBA5*	NM_024818.4	c.1111G>A	p.Ala371Thr	Het	Pathogenic	AR	Unknown	NA

Abbreviations: AD, autosomal dominant; AR, autosomal recessive; ES, exome sequencing; Hem, hemizygous; Het, heterozygous; NA, not applicable; VUS, variant of unknown significance; XL, X-linked; XLD, X-linked dominant.

^a^ Full list of genes in the microdeletion: *SEZ6L2*, *INO80E*, *SMG1P2*, *MIR3680-1*, *MIR3680-2*, *SPN*, *QPRT*, *C16orf54*,* ZG16*, *KIF22*, *MAZ*, *PRRT2*, *PAGR1*, *MVP*, *CDIPT*, *CDIPT-AS1*, *ASPHD1*, *KCTD13*, *TMEM219*, *TAOK2*, *HIRIP3*, *DOC2A*, *C16orf92*, *FAM57B*, *ALDOA*, *PPP4C*, T*BX6*, *YPEL3*, *LOC101928595*, *GDPD3*, *MAPK3*, *CORO1A*.

^b^ Microdeletion includes genes listed from *SPN* through *MAPK3* in footnote a.

^c^ Findings noted in medically actionable genes.

^d^ The report was marked as likely diagnostic owing to lack of definitive variant classification evidence and somewhat limited clinical validity of the gene.

### Targeted Epilepsy Panel Findings

**Figure 2.**
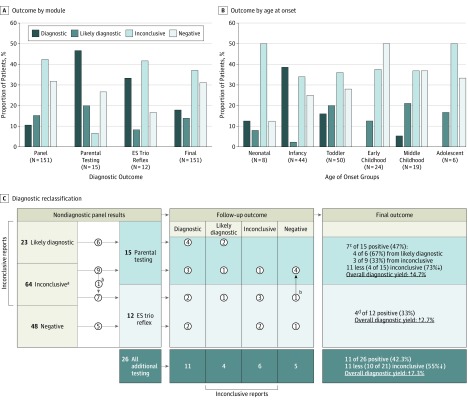
Diagnostic Outcomes A, Diagnostic outcome for different testing modules: proband-only panel, parental follow-up, exome sequencing (ES) trio reflex analysis, and overall. B, Diagnostic outcome by age at onset. C, Diagnostic reclassification by successive genetic testing. Numbers in circles indicate numbers of patients being retested. ^a^Parental testing indicated for 28 of 64 patients (43.8%). ^b^One patient was tested by both parental and ES trio follow-up and was also rendered negative. ^c^Parental testing revealed the apparent de novo nature of the variants. ^d^In genes not included in the panel.

Sixteen of the 151 patients (10.6%; 95% CI, 6%-16%) had diagnostic panel testing findings and therefore required no further testing (Figure 2A and Table 2). Recurrent findings included *SCN1A* (n = 3), *STXBP1* (n = 2), *IQSEC2* (n = 2), and chromosome 16p11.2 deletion syndrome (n = 2) (Table 2). Because the latter deletion includes the *PRRT2* gene, a total of 3 cases were diagnostic owing to findings involving this gene (2 deletions and 1 frameshift). Other diagnostic findings were in *ATP1A2*, *KCNQ2*, *KCNT1*, *PCDH19*, and *TPP1* genes. Of the 16 diagnostic results, 3 (18.8%) (2 in *PPRT2* and 1 in *DEPDC5*) had pathogenic CNVs detected using ES read-depth analysis and subsequently confirmed by orthogonal methods (concurrent diagnosis using CMA in patient 11). The exome-based CNV algorithm implemented added 2.0% (3 of 151) to the overall diagnostic yield.

Twenty-three patients (15.2%) had likely diagnostic findings for which parental testing was necessary to establish a definitive diagnosis (Figure 2A), that is, to determine whether the variants were de novo or establish phase (*cis* or *trans*) in the case of recessive genes. Testing in 64 patients (42.4%) yielded inconclusive findings. Of those, parental testing was indicated for 28 patients (43.8%), but not for the remaining 36 patients (56.3%), owing to limited variant evidence or the presence of a single heterozygous pathogenic variant in recessive genes. Forty-eight patients (31.8%) had no reportable findings (negative).

### Value of Follow-up Testing

Follow-up testing, consisting of parental testing (n = 15) or reflex trio exome analysis (n = 12), was performed for 26 of 135 patients (19.3%), with panel findings that were likely diagnostic in 6 patients (23.1%), inconclusive in 15 patients (57.8%), and negative in 5 patients (19.2%). One patient with panel-inconclusive findings underwent both parental follow-up and exome reflex. Although performed on a limited number of cases, parental and reflex exome testing found a causal variant in 11 of 26 studied cases (42.3%; 95% CI, 23%-62%), increasing the overall diagnostic yield by 7.3% (from 10.6% to 17.9%). Parental testing established 7 diagnosis (in *ATP1A3*, *CACNA1A*, *GABRA1*, *SCN1A*, *SCN2A*, *SCN8A*, and *UBE3A* genes), raising diagnostic yield by 4.7% to overall diagnostic yield of 15.3% (23 of 151; 95% CI, 9%-21%). Reflex exome analysis established 4 diagnosis (in *NR2F1*, *SETBP1*, *SMC1A*, and *TRIT1* genes), raising the diagnostic yield by 2.7% to overall diagnostic yield of 17.9% (27 of 151; 95% CI, 12%-24%).

Although parental testing was recommended for 23 likely diagnostic and 28 inconclusive probands, parental samples were received for only 6 likely diagnostic (26.1%) and 9 inconclusive (32.1%) probands. The recovery of samples for parental follow-up was only 29.4% (15 of 51). Diagnosis was established in 7 of 15 probands (46.7%; 95% CI, 21%-72%), of whom 4 had likely diagnostic and 3 had inconclusive panel findings (Figure 2C). Furthermore, inconclusive findings for 1 patient were upgraded to likely diagnostic, while negative results followed for 4 patients with panel-inconclusive findings. Altogether, parental testing decreased the inconclusive patient reports by 73% owing to reclassification of 11 (7 to diagnostic and 4 to negative reports).

Trio exome reflex testing was ordered for 7 patients with panel-inconclusive findings, 1 of whom had parental follow-up with negative outcome, and 5 patients with panel-negative results. This testing yielded a molecular diagnosis in 4 of 12 patients (33.3%; 95% CI, 6%-61%), of whom 2 had inconclusive panel findings and 2 had negative panel findings. The patient with panel-inconclusive findings followed by parental-negative results retained a negative result after ES trio. In addition, 1 patient with panel-inconclusive findings received a likely diagnostic result owing to the identification of de novo variants in 2 genes with limited clinical validity. Exome analysis rendered inconclusive findings for 2 patients with panel-negative results (Figure 2C).

We expect additional diagnostic findings as more nondiagnostic cases undergo follow-up testing. Although only 26.1% of patients with likely diagnostic panel findings underwent parental testing, 4 of them (66.7%) received diagnostic results.

### Diagnostic Yield Stratification

We investigated whether diagnostic yield varied by patient-specific attributes, namely, age at seizure onset, neurodevelopmental phenotype, seizure type, presence of magnetic resonance imaging findings, and family history (Table 1). The most striking stratification was observed with respect to age at seizure onset. The diagnostic yield was highest in infants (aged 1-12 months) (17 of 44 [38.6%; 95% CI, 24%-53%]) followed by toddlers (aged 12-36 months) (8 of 50 [16.0%; 95% CI, 6%-26%]), contributing 11% and 5%, respectively, to the overall diagnostic yield in the cohort (Table 1 and Figure 2B). There was only 1 diagnostic outcome (by exome trio reflex) in the neonates (aged 0-1 month) (1 of 8 [12.5%]), and 1 diagnosis in probands with seizure onset in late childhood (aged 6-11 years) (1 of 19 [5.3%]). No diagnoses were established in children aged 3 to 5 years (n = 24) or patients aged 12 to 18 years) (Table 1).

### Exome Reanalysis

The exome-based approach enabled us to reanalyze patients with negative and inconclusive results (n = 124) by opening variant analysis to genes that were associated with epilepsy after the test launch in 2016. Thirteen new genes (*CACNA1E*, *CACNA1I*, *CLCN4*, *GABBR2*, *KCNMA1*, *KCNQ5*, *MBD5*, *NUS1*, *SCN3A*, *SLC25A42*, *SLC2A1*, *SNAP25*, and *UBA5*) were included in this reanalysis. Almost all exons in those genes had adequate sequence coverage.^15^ Bioinformatics analysis identified 14 rare variants in these genes; however, on manual curation, only 6 variants in 6 patients were considered potentially significant (Table 2).

One heterozygous pathogenic variant was identified in *UBA5*, a gene with an autosomal recessive inheritance pattern, but a second sequence variant or CNV was not identified, making the result inconclusive. However, the presence of clinically significant noncoding variants undetectable by this test cannot be excluded.

In 5 other patients, heterozygous variants were identified in 4 autosomal dominant genes. The variants were rare or absent from the general population and affected highly conserved amino acid residues and/or functional domains. However, additional functional and/or segregation data are needed to establish the clinical significance of the variants. As more variant information becomes available for those newly discovered genes, we anticipate that some, if not all, of those variants will play significant roles in the patients’ seizures.

## Discussion

We have possibly validated and clinically implemented what may be a useful and flexible exome-based approach for genomic diagnostic testing of pediatric epilepsy. The overall diagnostic yield was 17.9% (27 of 151), which falls within the reported range by others (15%-28%).^21,22,23,24,25,26^ Diagnostic yield was significantly higher (38.6%) for infants (aged 1-12 months; 17 of 44) compared with other age groups, supporting the clinical use of this diagnostic approach in pediatric epilepsy, especially for infancy-onset seizures.

The initial yield after analysis of the 100 panel genes was 10.6%. Follow-up parental testing and trio exome reflex testing on a limited number of patients with nondiagnostic results (n = 26) increased the overall yield by 7.3% for a final rate of 17.9%. This is a significant increase given the limited number of patients who underwent follow-up testing (26 of 135 [19.3%]). Seven of 15 patients (46.7%) who underwent parental follow-up testing and 4 of 12 patients (33.3%) who underwent exome reflex received diagnoses. Parental follow-up established diagnosis for 4 of 6 of the probands (66.7%) with initially likely diagnostic findings and 3 of 9 probands (33.3%) with initially inconclusive findings (Figure 2C). Determining the inheritance of the variants was necessary for establishing the molecular diagnoses in these probands. In addition, 4 of 15 (26.7%) of the patients received negative reports at parental follow-up, which decreased the inconclusive patient reports by 73% (7 diagnostic and 4 negative of 15 tested). Exome reflex uncovered diagnostic findings for 4 probands.

In addition to being a useful testing option, this exome-based approach may enable flexible update of panel gene content with minimal test validation to keep up with the continuous discovery of new epilepsy genes, and also allows periodic reanalysis on patients with nondiagnostic results. Reanalysis was performed on a limited number of genes (n = 13) for all patients who did not receive a molecular diagnosis within 1 year after initial testing (n = 124). Although this analysis uncovered promising findings in 6 patients, reanalysis on a larger number of genes and for an extended time (>2 years after initial testing) might be more useful in identifying additional diagnoses.

The study findings are in agreement with recent reports that sequencing-based diagnostics is effective as first-tier testing in pediatric epilepsy.^21,27^ Moreover, integration of copy number calling from ES makes the testing more comprehensive and increases cost-effectiveness by eliminating the necessity of CMA testing. The exome-based CNV algorithm implemented here identified pathogenic variants in 3 of 27 of the positive cases (11.1%) adding 2.0% (3 of 151) to the overall diagnostic yield.

**Figure 3.**
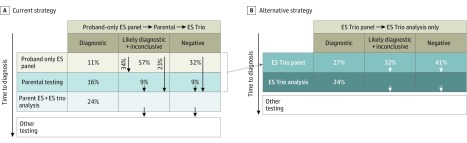
Current and Alternative Strategies for Genetic Testing in Childhood Epilepsy A, Current strategy: proband-only exome sequencing (ES) followed by parental testing of select patients with inconclusive results and reflex to ES trio testing of the remaining patients with nondiagnostic results. B, Alternative trio ES panel strategy: starts with ES trio (proband plus parents) panel analysis followed by immediate reflex to ES analysis for trios with nondiagnostic panel results. The percentage of cases at each testing stage is extrapolated to the full cohort using this study’s data. Test costs are in reference to proband-only ES panel as the base cost (relative cost: proband-only ES panel: X; trio ES panel: 1.7X; ES trio testing: 2.5X; overall cohort cost: strategy B is less than 10% higher than strategy A).

Based on this study, we recommend that parental samples be obtained simultaneously with the proband’s because parental samples are necessary for interpretation of identified variants. We recommend that proband-only exome-based rapid analysis of a preselected set of genes should be the first step, followed by parental testing of select inconclusive findings and then ES trio testing of the remaining patients with nondiagnostic results (Figure 3A).

Alternatively, a strategy in which initial testing includes an ES panel on a family trio (parents and proband) could be considered (Figure 3B). This strategy would eliminate the need for follow-up parental testing and would increase the confidence for de novo findings by providing the certainty for a biological relationship, which would enable immediate reflex to whole-exome analysis and decrease the time to diagnosis. A disadvantage of this panel trio strategy, however, would be the unnecessary cost of parental ES for 11% of patients with diagnostic panel results. Extrapolating from parental follow-up data in Figure 2C, parental testing could reveal diagnosis in an additional 16% of the patients, and reflex to ES trio would be indicated for the remaining 73% of probands (Figure 3A). Based on these numbers, the cost of the 2 strategies is comparable, with the panel trio strategy adding less than 10% additional costs (Figure 3B). This is because the cost of testing is driven by analysis and interpretation costs rather than sequencing cost, with the former accounting for approximately 70% for proband-only ES and 60% for ES trio testing. Despite the increase in sequencing costs for 27% of the probands, the analysis/interpretation cost would be significantly reduced (owing to automated inheritance modeling), which would partially offset the increase in sequencing costs. In addition, this moderate increase in testing cost might be worthwhile given the health care benefits of the shortened time to diagnosis, which is not only associated with reduced overall cost of the patient's diagnostic workup, but would also be of significance for delivering timely, personalized treatments.

### Limitations

The main limitations of this study include a relatively small sample size, possible selection bias of patients who received testing, and significant loss of samples to parental and ES reflex follow-up. Clinicians’ decisions on which patients would benefit from this test could cause patient selection bias, which would lead to an overestimation of contribution of parental and ES follow-up testing in assessing overall diagnostic yield. The recovery of samples for parental follow-up was only 29.4% (15 of 51), which could be attributable to parental availability, as parental testing was free-of-charge. However, availability of insurance coverage may have contributed to the reduced numbers of reflex to ES trio analysis.

## Conclusions

Most epilepsy genetic testing is based on a proband-only 1-and-done strategy. This case series report suggests that both parental testing and reflex to ES are important for maximizing diagnostic yield. The study also supports the importance of regular reanalysis efforts to incorporate newly discovered epilepsy gene evaluation and demonstrates exome-based panels as a readily amenable option for this purpose.

## Notes

Supplement. **eMethods 1.** Sequence Coverage Assessment, Sanger Sequencing Fill-Ins and CNV Confirmation **eMethods 2.** Rapid Epilepsy Sequence and Deletion/Duplication (STAT) Panel **eReference.** **eTable.** Gene Identification Numbers

## References

[1] Fiest KM, Sauro KM, Wiebe S, Prevalence and incidence of epilepsy: a systematic review and meta-analysis of international studies. Neurology. 2017;88(3):-. doi:10.1212/WNL.0000000000003509 27986877 PMC5272794

[2] Myers CT, Mefford HC Advancing epilepsy genetics in the genomic era. Genome Med. 2015;7:91. doi:10.1186/s13073-015-0214-7 26302787 PMC4549122

[3] Thomas RH, Berkovic SF The hidden genetics of epilepsy—a clinically important new paradigm. Nat Rev Neurol. 2014;10(5):283-292. doi:10.1038/nrneurol.2014.62 24733163

[4] McTague A, Howell KB, Cross JH, Kurian MA, Scheffer IE The genetic landscape of the epileptic encephalopathies of infancy and childhood. Lancet Neurol. 2016;15(3):304-316. doi:10.1016/S1474-4422(15)00250-1 26597089

[5] Nicita F, De Liso P, Danti FR, The genetics of monogenic idiopathic epilepsies and epileptic encephalopathies. Seizure. 2012;21(1):3-11. doi:10.1016/j.seizure.2011.08.007 21917483

[6] Sands TT, Choi H Genetic testing in pediatric epilepsy. Curr Neurol Neurosci Rep. 2017;17(5):45. doi:10.1007/s11910-017-0753-y 28405954

[7] Szepetowski P Genetics of human epilepsies: continuing progress. Presse Med. 2018;47(3):218-226. doi:10.1016/j.lpm.2017.10.020 29277263

[8] Wang J, Lin ZJ, Liu L, Epilepsy-associated genes. Seizure. 2017;44:11-20. doi:10.1016/j.seizure.2016.11.030 28007376

[9] Helbig I, Tayoun AA Understanding genotypes and phenotypes in epileptic encephalopathies. Mol Syndromol. 2016;7(4):172-181. doi:10.1159/000448530 27781027 PMC5073622

[10] Dunn P, Albury CL, Maksemous N, Next generation sequencing methods for diagnosis of epilepsy syndromes. Front Genet. 2018;9:20. doi:10.3389/fgene.2018.00020 29467791 PMC5808353

[11] Orsini A, Zara F, Striano P Recent advances in epilepsy genetics. Neurosci Lett. 2018;667:4-9. doi:10.1016/j.neulet.2017.05.014 28499889

[12] Strande NT, Riggs ER, Buchanan AH, Evaluating the clinical validity of gene-disease associations: an evidence-based framework developed by the Clinical Genome Resource. Am J Hum Genet. 2017;100(6):895-906. doi:10.1016/j.ajhg.2017.04.015 28552198 PMC5473734

[13] Helbig I, Riggs ER, Barry CA, The ClinGen Epilepsy Gene Curation Expert Panel—bridging the divide between clinical domain knowledge and formal gene curation criteria. Hum Mutat. 2018;39(11):1476-1484. doi:10.1002/humu.23632 30311377 PMC7418072

[14] Guan Q, Balciuniene J, Cao K, AUDIOME: a tiered exome sequencing–based comprehensive gene panel for the diagnosis of heterogeneous nonsyndromic sensorineural hearing loss. Genet Med. 2018;20(12):1600-1608. doi:10.1038/gim.2018.48 29595809

[15] Niazi R, Gonzalez MA, Balciuniene J, Evans P, Sarmady M, Abou Tayoun AN The development and validation of clinical exome-based panels using ExomeSlicer: considerations and proof of concept using an epilepsy panel. J Mol Diagn. 2018;20(5):643-652. doi:10.1016/j.jmoldx.2018.05.003 29936260

[16] Plagnol V, Curtis J, Epstein M, A robust model for read count data in exome sequencing experiments and implications for copy number variant calling. Bioinformatics. 2012;28(21):2747-2754. doi:10.1093/bioinformatics/bts526 22942019 PMC3476336

[17] Richards S, Aziz N, Bale S, ; ACMG Laboratory Quality Assurance Committee Standards and guidelines for the interpretation of sequence variants: a joint consensus recommendation of the American College of Medical Genetics and Genomics and the Association for Molecular Pathology. Genet Med. 2015;17(5):405-424. doi:10.1038/gim.2015.30 25741868 PMC4544753

[18] BioRxiv. Variation across 141,456 human exomes and genomes reveals the spectrum of loss-of-function intolerance across human protein-coding genes. https://www.biorxiv.org/content/10.1101/531210v2. Published January 30, 2019*. *Accessed February 15, 2019.

[19] Preacher KJ Calculation for the chi-square test: an interactive calculation tool for chi-square tests of goodness of fit and independence. http://www.quantpsy.org/chisq/chisq.htm. Published April 2001. Accessed February 15, 2019.

[20] Romasko EJ, DeChene ET, Balciuniene J, PCDH19-related epilepsy in a male with Klinefelter syndrome: additional evidence supporting *PCDH19* cellular interference disease mechanism. Epilepsy Res. 2018;145:89-92. doi:10.1016/j.eplepsyres.2018.06.008 29933145

[21] Berg AT, Coryell J, Saneto RP, Early-life epilepsies and the emerging role of genetic testing. JAMA Pediatr. 2017;171(9):863-871. doi:10.1001/jamapediatrics.2017.1743 28759667 PMC5710404

[22] Butler KM, da Silva C, Alexander JJ, Hegde M, Escayg A Diagnostic yield from 339 epilepsy patients screened on a clinical gene panel. Pediatr Neurol. 2017;77:61-66. doi:10.1016/j.pediatrneurol.2017.09.003 29056246 PMC6885003

[23] Kothur K, Holman K, Farnsworth E, Diagnostic yield of targeted massively parallel sequencing in children with epileptic encephalopathy. Seizure. 2018;59:132-140. doi:10.1016/j.seizure.2018.05.005 29852413

[24] Lindy AS, Stosser MB, Butler E, Diagnostic outcomes for genetic testing of 70 genes in 8565 patients with epilepsy and neurodevelopmental disorders. Epilepsia. 2018;59(5):1062-1071. doi:10.1111/epi.14074 29655203

[25] Ortega-Moreno L, Giráldez BG, Soto-Insuga V, ; Grupo Español de Genética de las Epilepsias de la Infancia (GEGEI) Molecular diagnosis of patients with epilepsy and developmental delay using a customized panel of epilepsy genes. PLoS One. 2017;12(11):e0188978. doi:10.1371/journal.pone.0188978 29190809 PMC5708701

[26] Staněk D, Laššuthová P, Štěrbová K, Detection rate of causal variants in severe childhood epilepsy is highest in patients with seizure onset within the first four weeks of life. Orphanet J Rare Dis. 2018;13(1):71. doi:10.1186/s13023-018-0812-8 29720203 PMC5932755

[27] Myers KA, Johnstone DL, Dyment DA Epilepsy genetics: current knowledge, applications, and future directions. Clin Genet. 2019;95(1):95-111. doi:10.1111/cge.13414 29992546

