# Supplementary material for: Use of a Dynamic Genetic Testing Approach for Childhood-Onset Epilepsy
Source: JAMA Netw Open. 2019 Apr 12;2(4):e192129. doi: 10.1001/jamanetworkopen.2019.2129 (PMC6481455; doi:10.1001/jamanetworkopen.2019.2129)
Supplement: Supplement. — eMethods 1. Sequence Coverage Assessment, Sanger Sequencing Fill-Ins and CNV Confirmation eMethods 2. Rapid Epilepsy Sequence and Deletion/Duplication (STAT) Panel eReference. eTable. Gene Identification Numbers [file jamanetwopen-2-e192129-s001.pdf]

Supplementary Online Content

Balciuniene J, DeChene ET, Akgumus G, et al. Use of a dynamic genetic testing approach for childhood-onset epilepsy. *JAMA Netw Open*. 2019;2(4):e192129. doi:10.1001/jamanetworkopen.2019.2129

eMethods 1. Sequence Coverage Assessment, Sanger Sequencing Fill-Ins and CNV Confirmation

eMethods 2. Rapid Epilepsy Sequence and Deletion/Duplication (STAT) Panel

eReference.

eTable. Gene Identification Numbers

This supplementary material has been provided by the authors to give readers additional information about their work.

© 2019 Balciuniene J et al. *JAMA Network Open.*

eMethods 1. Sequence Coverage Assessment, Sanger Sequencing Fill-Ins and CNV Confirmation

The sequence coverage of the exonic sequences (±15 intronic bp) of the 100 panel genes was assessed using the BamStats04 tool and GATK DepthOfCoverage. A base pair with at least 10 sequencing reads was considered "fully" covered. Exons with 10 or more consecutive bases covered by <10X were filled-in using Sanger sequencing[1]. Sanger sequencing was also used for technically challenging regions (polyalanine tract expansion in *ARX*) and to confirm all pathogenic and likely pathogenic variants, and variants of uncertain significance (VUS) with low quality metrics as determined by the bioinformatics pipeline. CNVs involving the epilepsy panel genes were confirmed by droplet digital PCR (ddPCR) or another methodology before reporting. CNVs that did not include an epilepsy panel gene were filtered out by the pipeline.

eMethods 2. Rapid Epilepsy Sequence and Deletion/Duplication (STAT) Panel

The rapid epilepsy panel includes sequencing and copy number analysis of 19 epilepsy-associated medically actionable genes. Coding regions and splice sites of those genes were captured using the SureSelectQXT Target Enrichment System (Agilent Inc) followed by NGS. Further processing of the NGS data was performed using in-house bioinformatics pipeline that uses the same tools as described above.
Copy number analysis for those genes (except *GRIN2D* and *KCNT1*) was performed using targeted analysis from a custom comparative genomic hybridization array (aCGH) (Agilent Technologies) following the manufacturer's protocol. The array includes 4 probes on average for each targeted exon and one probe every 30 kb throughout the rest of the genome.

Genes included in this panel are: *ALDH7A1, CSTB, EPM2A, GAMT, GATM, GRIN2A, GRIN2D, GRIN2B, KCNB1, KCNQ2, KCNT1, NHLRC1, PNPO, POLG, SCN1A, SCN2A, SCN8A, SLC2A1, SLC6A8.*

eReference

1. Niazi R, Gonzalez MA, Balciuniene J, Evans P, Sarmady M, Abou Tayoun AN. The Development and Validation of Clinical Exome-Based Panels Using ExomeSlicer: Considerations and Proof of Concept Using an Epilepsy Panel. *J Mol Diagn.* 2018;20(5):643-652.

© 2019 Balciuniene J et al. *JAMA Network Open.*

eTable. Gene Identification Numbers

CHOP epilepsy panel genes with OMIM IDs

Gene Symbol	OMIM ID
ALDH7A1	107323
ALG13	300776
ARHGEF9	300429
ARX	300382
ASAH1	613468
ATP1A2	182340
ATP1A3	182350
CACNA1A	601011
CACNA1D	114206
CASK	300172
CDKL5	300203
CERS1	606919
CHD2	602119
CHRNA2	118502
CHRNA4	118504
CHRNB2	118507
CLN3	607042
CLN5	608102
CLN6	606725
CLN8	607837
CNKSR2	300724
CSTB	601145
CTSD	116840
DEPDC5	614191
DNM1	602377
DYNC1H1	600112
EEF1A2	602959
EFHC1	608815
EPM2A	607566
FOLR1	136430
FOXG1	164874
GABRA1	137160
GABRB3	137192
GABRG2	137164
GNAO1	139311
GOSR2	604027

© 2019 Balciuniene J et al. *JAMA Network Open.*

GRIN1	138249
GRIN2A	138253
GRIN2B	138252
HCN1	602780
HDAC4	605314
HNRNPU	602869
IQSEC2	300522
KCNA1	176260
KCNA2	176262
KCNB1	600397
KCNC1	176258
KCNJ10	602208
KCNQ2	602235
KCNQ3	602232
KCNT1	608167
KCTD7	611725
LGI1	604619
MECP2	300005
MEF2C	600662
MFSD8	611124
NHLRC1	608072
PCDH19	300460
PIGA	311770
PIGO	614730
PIGT	610272
PLCB1	607120
PNKP	605610
PNPO	603287
POLG	174763
PPT1	600722
PRICKLE1	608500
PRRT2	614386
PURA	600473
QARS	603727
RELN	600514
RYR3	180903
SCARB2	602257
SCN1A	182389
SCN1B	600235
SCN2A	182390
SCN8A	600702

© 2019 Balciuniene J et al. *JAMA Network Open.*

SIK1	605705
SLC12A5	606726
SLC13A5	608305
SLC25A22	609302
SLC2A1	138140
SLC35A2	314375
SLC6A1	137165
SLC6A8	300036
SPTAN1	182810
ST3GAL3	606494
STX1B	601485
STXBP1	602926
SYN1	313440
SYNGAP1	603384
SZT2	615463
TBC1D24	613577
TPP1	607998
TSC1	605284
TSC2	191092
UBE3A	601623
WDR45	300526
WWOX	605131
ZEB2	605802

© 2019 Balciuniene J et al. *JAMA Network Open.*
